# Translational roles of the C75 2′OH in an *in vitro* tRNA transcript at the ribosomal A, P and E sites

**DOI:** 10.1038/s41598-017-06991-6

**Published:** 2017-07-27

**Authors:** Jinfan Wang, Anthony C. Forster

**Affiliations:** Department of Cell and Molecular Biology, Uppsala University, Husargatan 3, Box 596, Uppsala, 75124 Sweden

## Abstract

Aminoacyl-tRNAs containing a deoxy substitution in the penultimate nucleotide (C75 2′OH → 2′H) have been widely used in translation for incorporation of unnatural amino acids (AAs). However, this supposedly innocuous modification surprisingly increased peptidyl-tRNA^Ala^_ugc_ drop off in biochemical assays of successive incorporations. Here we predict the function of this tRNA 2′OH in the ribosomal A, P and E sites using recent co-crystal structures of ribosomes and tRNA substrates and test these structure-function models by systematic kinetics analyses. Unexpectedly, the C75 2′H did not affect A- to P-site translocation nor peptidyl donor activity of tRNA^Ala^_ugc_. Rather, the peptidyl acceptor activity of the A-site Ala-tRNA^Ala^_ugc_ and the translocation of the P-site deacylated tRNA^Ala^_ugc_ to the E site were impeded. Delivery by EF-Tu was not significantly affected. This broadens our view of the roles of 2′OH groups in tRNAs in translation.

## Introduction

The 2′-hydroxyl group (2′OH) of the ribose in the backbone of ribonucleic acids (RNAs) is the main difference between RNAs and deoxyribonucleic acids (DNAs). Because of the presence of the 2′OH in RNAs, the sugar pucker is predominantly in the C3′-endo conformation in contrast to the C2′-endo conformation that predominates in DNAs^1^. Importantly, the 2′OH can be involved in intra- and inter-molecular hydrogen bonding interactions to contribute to the structure and function of RNA.

RNAs play crucial roles in protein synthesis where the macromolecular translation machinery, the ribosome, is an RNA-protein complex^2^. The ribosome catalyzes the polymerization of amino acids (AAs) in the sequence encoded by the messenger RNA (mRNA); the site in the ribosome where peptide bond formation occurs, the peptidyl transferase center (PTC), is composed entirely of RNA; AAs are linked to transfer RNAs (tRNAs) via ester bonds with the terminal A76 of the tRNAs before they can serve as the substrates of ribosomes^2^.

The roles of the 2′OH groups in tRNA bodies have been studied in different translation steps. For example, Feinberg and Joseph identified two 2′OH groups, at positions 71 and 76 of tRNA, that were essential for translocation of the P-site tRNA to the E site, and these two 2′OH groups were shown to contact the backbone of 23S rRNA in the E site in the crystal structure of the ribosome^3^. Pleiss and Uhlenbeck found that five distinct 2′OH groups in unmodified yeast tRNA^Phe^ were critical for the binding affinity of the AA-tRNA to elongation factor Tu (EF-Tu)^4^, noting that prior co-crystallographic data showed that four of these 2′OH groups formed inter-molecular hydrogen bonds with EF-Tu and the fifth formed an intra-molecular hydrogen bond. Interestingly, though EF-Tu recognizes the AA and A76 terminal 2′OH of the AA-tRNA, this 2′OH and the neighboring C75 penultimate 2′OH were shown to be unimportant for EF-Tu binding^4^. However, another study showed that replacing the A76 terminal 2′OH with 2′H abolished binding between a different AA-tRNA and EF-Tu^5^. This latter study also demonstrated important roles of this terminal 2′OH in peptidyl transfer and peptide release reactions, presumably by orienting the substrate for catalysis and/or facilitating proton transfer in the catalysis.

The 2′OH in the penultimate C75 of tRNAs, however, was believed to be unimportant in translation. AA-tRNAs containing a 2′H in C75, created by ligation of the 3′CA-truncated unmodified tRNAs to pdCpA-amino acids, had been widely used in translation for incorporation of unnatural AAs^6–8^. The lack of modifications and the C75 2′H in the tRNA body showed negligible effect on single incorporation yield of an AA into polypeptide^8–10^, and therefore such changes were believed to play only minor﻿ roles in translation. Surprisingly, when incorporating multiple AAs consecutively with the unmodified AA-tRNA^Ala^_ugc_ bearing the 2′H in C75, we found that these supposedly benign changes caused a big reduction in full-length peptide yields^10^ by allowing more time for peptidyl-tRNA drop off^11^. This reduction was not due to the lack of tRNA modifications as the kinetics and yields with unmodified C75 2′ OH transcript were indistinguishable from the native modified isoacceptor^10, 11^, despite studies showing critical roles of tRNA modifications in some other tRNAs^12–17^. Here we use recent ribosome-substrate crystal structures to predict the function of the 2′OH in C75 of the A-, P- and E-site tRNA^Ala^_ugc_ and test these structure-function models by fast kinetics.

## Results and Discussion

### Interactions of the tRNA C75 2′OH in ribosomal A-, P- and E-sites

**Figure 1:**
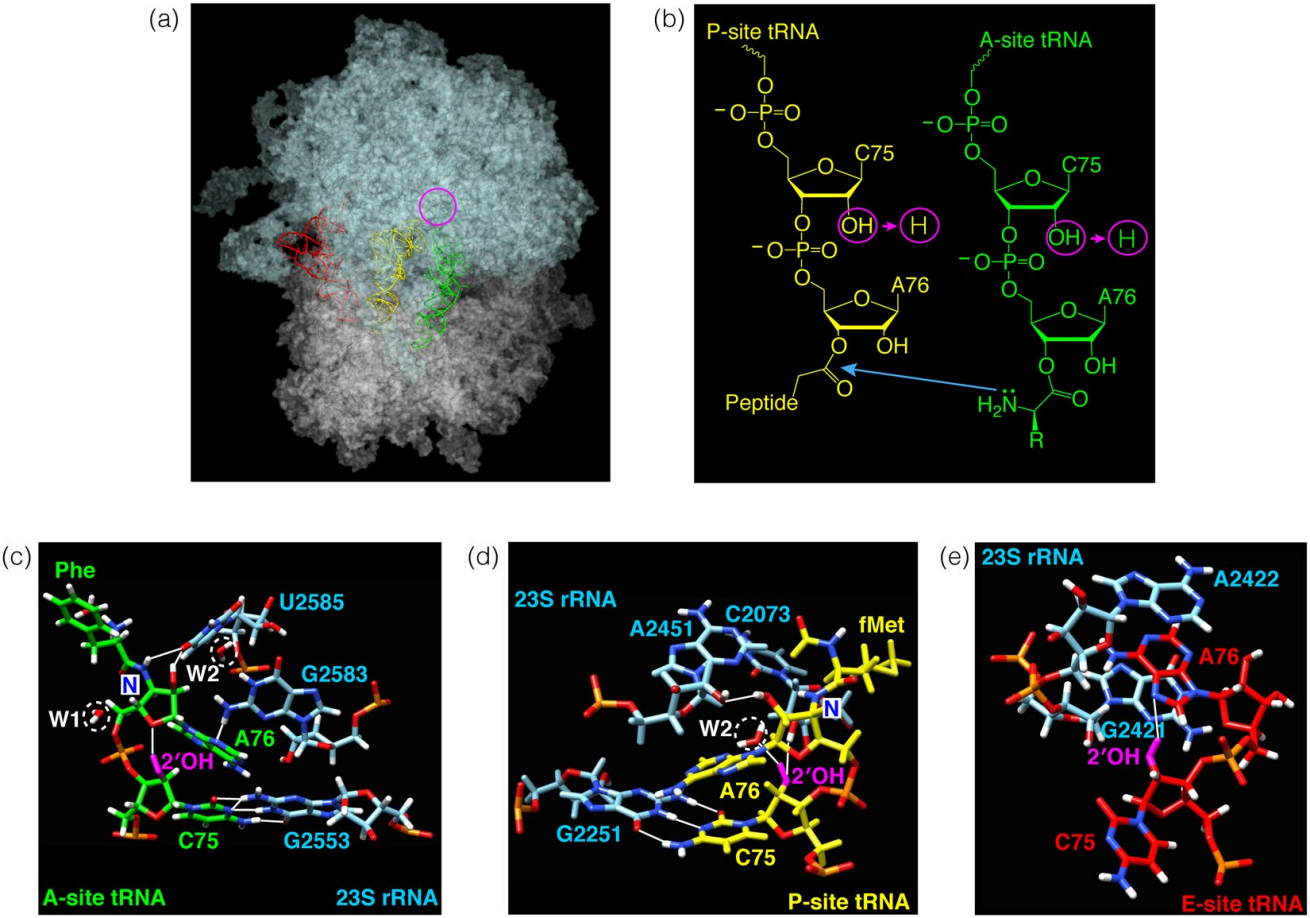
Interactions of the tRNA C75 2′OH in ribosomal A-, P- and E-sites in crystal structures. (**a**) tRNA substrates at the aminoacyl (green), peptidyl (yellow) and exit (red) sites on the 70S ribosome in 3 dimensions. The PTC is circled. (**b**) Two-dimensional chemical structures showing the covalent bonds separating the C75 2′OH groups (circled) from the nucleophile and electrophile of the peptidyl transfer reaction (blue arrow). (**c–e**) Crystal structures of the A-, P- and E-site tRNA substrates, respectively. Models were constructed with UCSF Chimera and PDB file 1VY4^18^. In the original PDB file, three water molecules found within the PTC were proposed to be essential for forming a “proton wire” to couple AA-tRNA accommodation and peptide bond formation. Two of these water molecules are ringed with white dashed circles and labeled W1 and W2 (W3 is not visible in this view). H-bonds are shown with white thin lines. (**c**) A-site Phe-tRNA^Phe^ in green, with the 2′OH in magenta. C75 forms a Watson-Crick base pair with G2553 of 23S rRNA, and the 2′OH of C75 forms an H bond with the O4 of the A76 sugar pucker. N indicates that Phe was linked to the tRNA with an amide bond. (**d**) P-site fMet-tRNA^fMet^ in yellow, with the 2′OH in magenta. C75 forms a Watson-Crick base pair with G2251 of 23S rRNA, and the 2′OH of C75 forms H bonds with W2 and the 2′OH of ribose C2073 of 23S rRNA. N indicates that fMet was linked to the tRNA with an amide bond. (**e**) E-site tRNA^Phe^ in red, with the 2′OH in magenta. C75 does not form a base pair, whereas the 2′OH of C75 forms an H-bond with the N7 of the base of A76 in the same tRNA.

An important test of our understanding of the relationship of structure to function is to be able to predict the functional effects of mutants, or failing that, at least rationalize the results obtained. In an attempt to predict which translation step(s) are affected by the dC75 atomic mutation in tRNA^Ala^_ugc_, we examined recent co-crystal structures of the 70S ribosome bound to substrate analogs^18^. These structures show substrates in A-, P- and E-sites (Fig. 1a) and are of sufficiently high resolution around the critical C75 2′OH (Fig. 1b) to show water molecules (Fig. 1c,d).

Given that the C75 2′OH forms H bonds at the A, P and E sites, it is easy to rationalize our overall inhibition of translation observed by replacement with a 2′H in these 3 positions on the ribosome. The loss of the A-site H bond to the O4 of the A76 sugar, which in turn is attached to the AA nucleophile, may be predicted to affect correct positioning for nucleophilic attack. The loss of the P-site H bonds with W2 and with the 2′OH of ribose C2073 of 23S rRNA may be predicted to affect correct positioning of the electrophile and also translocation of the A site peptidyl-tRNA to the P site (because the A site tRNA would not form the H-bond with the P site rRNA). The loss of the E-site intramolecular H-bond (Fig. 1e) might be less important, so P to E site translocation may be predicted to be unaffected.

### The C75 2′H impedes the peptidyl acceptor activity of Ala-tRNA^Ala^ but not delivery by EF-Tu

**Figure 2:**
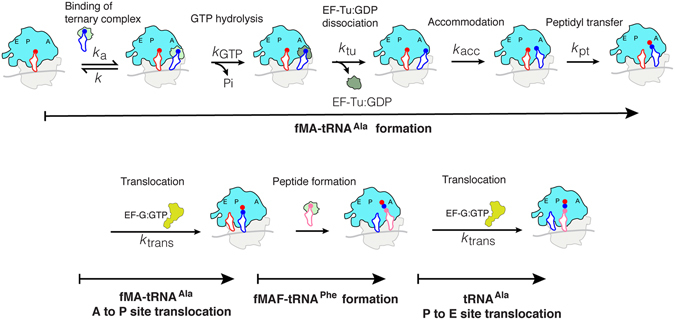
Schematic illustration of the kinetic steps in tripeptide synthesis. The EF-Tu:GTP:Ala-tRNA^Ala^ ternary complex was added to the 70S initiation complex and the mean time for GTP hydrolysis on EF-Tu was measured. After GTP hydrolysis and ET-Tu:GDP was dissociated from the ribosome, the Ala-tRNA^Ala^ was accommodated to accept the fMet to form the fMA dipeptide. The mean time for fMA-tRNA^Ala^ formation was measured. After the formation of fMA-tRNA^Ala^, this dipeptidyl-tRNA was translocated from the A site to the P site by EF-G and could then donate the fMA dipeptide to the incoming A site Phe-tRNA^Phe^ to form the fMAF tripeptide. The resulting P site deacylated tRNA^Ala^ was translocated to the E site along with the peptidyl-tRNA^Phe^ translocating to the P site.

To test our predictions based on crystal structures, we prepared Ala-tRNA^Ala^ substrates with either C75 2′OH (Ala-${{\rm{tRNA}}}_{{\rm{ugc}}}^{{\rm{AlaB}}}\_{\rm{enzymatic}}$) or 2′H (Ala-${{\rm{tRNA}}}_{{\rm{ugc}}}^{{\rm{AlaB}}}\_{\rm{dC}}$) (Supplementary Figure 1). Ala-${{\rm{tRNA}}}_{{\rm{ugc}}}^{{\rm{AlaB}}}\_{\rm{enzymatic}}$ was prepared from *in vitro* transcribed full-length tRNA^Ala^ by charging with Ala using the AA-tRNA synthetase AlaRS. Ala-${{\rm{tRNA}}}_{{\rm{ugc}}}^{{\rm{AlaB}}}\_{\rm{dC}}$ was prepared by ligating *in vitro*-transcribed 3′CA-truncated tRNA^Ala^ to *N*-NVOC-Ala-pdCpA followed by photolytic removal of the NVOC group. We measured the following successive individual translation steps, listed in the order in which they occur in translation (Fig. 2): GTP hydrolysis on EF-Tu (to assay delivery of Ala-tRNA^Ala^ to the ribosomal A site), the subsequent time for fMet-Ala (fMA) dipeptide bond formation (to assay peptidyl acceptor activity of Ala-tRNA^Ala^), A to P site translocation of fMA-tRNA^Ala^, fMet-Ala-Phe (fMAF) tripeptide formation from EF-Tu:GTP:Phe-tRNA^Phe^ and fMA-tRNA^Ala^ (to assay peptidyl donor activity of P site fMA-tRNA^Ala^), and P to E site translocation of tRNA^Ala^.

**Figure 3:**
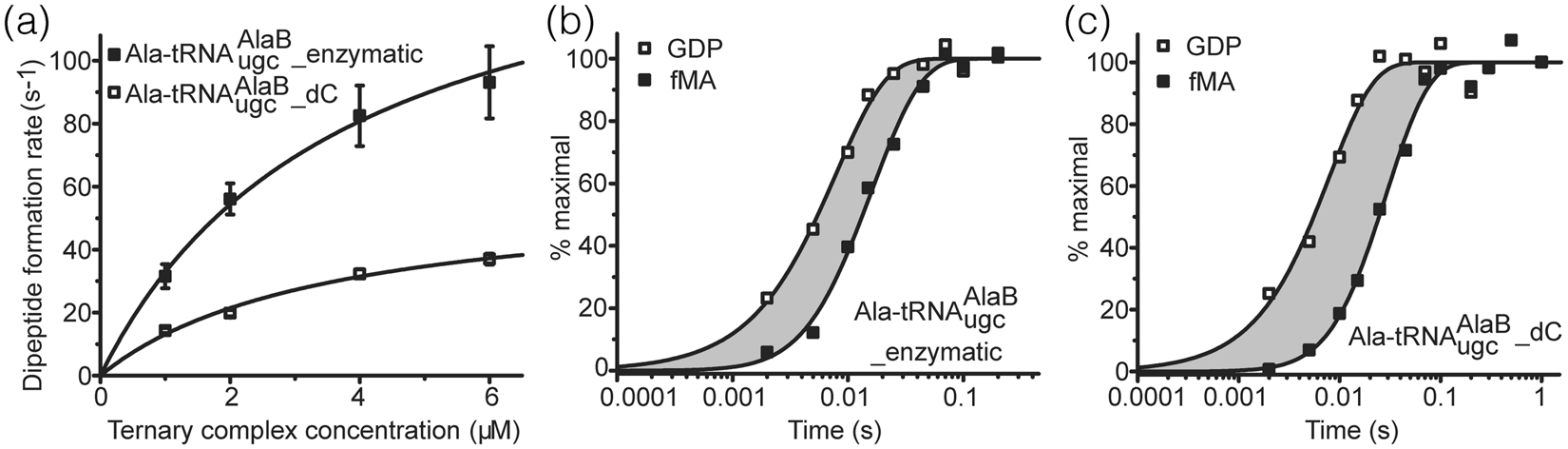
Kinetics of fMA dipeptide formation from fMet-tRNA^fMet^ and Ala-tRNAs. (**a**) Plot of the rates of dipeptide formation versus ternary complex concentrations. Mean values and standard deviations were calculated from at least two independent experiments (see Supplementary Table 1 and Supplementary Figure 2). The smooth curves show the fitting of the data to a hyperbolic Michaelis-Menten equation. (**b**) and (**c**) Plots of representative normalized time-evolution of GTP hydrolysis and dipeptide formation versus time for reactions with Ala-tRNA^AlaB^_ugc__enzymatic (**b**) and Ala-tRNA^AlaB^_ugc__dC (**c**). The shaded areas show the overall mean time of passage from the A/T state after GTP hydrolysis on EF-Tu to peptidyl transfer, *τ*_*acc,pep*_ (see Table 1). Experiments were conducted at least in duplicates.

Initially, we measured the rates of fMA dipeptide formation at different EF-Tu:GTP:Ala-tRNA^Ala^ ternary complex concentrations of the two kinds of substrates (Supplementary Table 1). Fitting these rates to a hyperbolic Michaelis-Menten equation allowed the estimation of the maximal overall rate of fMA dipeptide formation, *k*_cat_, and the *K*_M_ values (Fig. 3a and Table 1). *k*_cat_ for Ala-${{\rm{tRNA}}}_{{\rm{ugc}}}^{{\rm{AlaB}}}\_{\rm{dC}}$ was 59 ± 4 s^−1^ whereas for Ala-${{\rm{tRNA}}}_{{\rm{ugc}}}^{{\rm{AlaB}}}\_{\rm{enzymatic}}$ it was significantly faster at 157 ± 37 s^−1^, but the respective *K*_*M*_ values were similar (3.5 ± 0.5 μM and 3.8 ± 1.4 μM). This indicated that one or more steps up to the peptidyl transfer reaction were impeded by replacing the C75 2′OH with the 2′H. Next, we measured the mean times for GTP hydrolysis on EF-Tu (*τ*_*GTP*_) and dipeptide formation (*τ*_*dip*_) simultaneously (Fig. 3b,c and Table 1). *τ*_*GTP*_ for the two cases was the same, ~8 msec, so the delivery of the AA-tRNA to the ribosomal A site by EF-Tu:GTP was not impeded by the lack of the C75 2′OH. However, the time for dipeptide formation, *τ*_*dip*_, with Ala-${{\rm{tRNA}}}_{{\rm{ugc}}}^{{\rm{AlaB}}}\_{\rm{dC}}$ (~33 msec) was about two-fold longer than that of Ala-${{\rm{tRNA}}}_{{\rm{ugc}}}^{{\rm{AlaB}}}\_{\rm{enzymatic}}$ (~18 msec). This indicated impeded (i) release of the 3′CCA end of the tRNA from EF-Tu:GDP, (ii) AA-tRNA accommodation, and/or (iii) peptidyl transfer reaction. The overall mean time for these three steps, *τ*_*acc,pep*_, could be calculated by subtracting *τ*_*GTP*_ from *τ*_*dip*_ (Table 1), but independently measuring these three steps is not tractable. Although an impeded release by EF-Tu-GDP or accommodation step cannot be ruled out, we speculate that it is the peptidyl transfer reaction that is affected by the C75 2′H. The conformation of peptidyl transferase center is induced by base pairing of the A-site tRNA penultimate C with the G2553 of the 23S rRNA, and proper orienting of the ester link of the P-site tRNA is important for peptidyl transfer^19–24^. Replacing the penultimate C with dC might change the ribonucleotide-favored C3′-*endo* sugar conformation to a deoxyribonucleotide-favored C2′-*endo* sugar conformation in the tRNA^25^. The result also supports our prediction that the loss of the A-site H bond to the O4 of the A76 sugar, which in turn is attached to the AA nucleophile, may affect correct positioning for nucleophilic attack.

**Table 1:** Kinetic parameters for fMA dipeptide formation (see Fig. 3).

AA-tRNA	*k*_*cat*_ (s^−1^)	*K*_*M*_ (μM)	*τ*_*GTP*_ (msec)	*τ*_*dip*_ (msec)	*τ*_*acc,pep*_ (msec)
Ala-${{\rm{tRNA}}}_{{\rm{ugc}}}^{{\rm{AlaB}}}\_{\rm{dC}}$	59 ± 4	3.5 ± 0.5	8 ± 1	33 ± 3	25 ± 3
Ala-${{\rm{tRNA}}}_{{\rm{ugc}}}^{{\rm{AlaB}}}\_{\rm{enzymatic}}$	157 ± 37	3.8 ± 1.4	8 ± 1	18 ± 2	10 ± 2

Mean values were calculated from at least two independent experimental results with their propagated standard deviations.

### The C75 2′H does not affect A- to P-site translocation nor peptidyl donor activity

**Figure 4:**
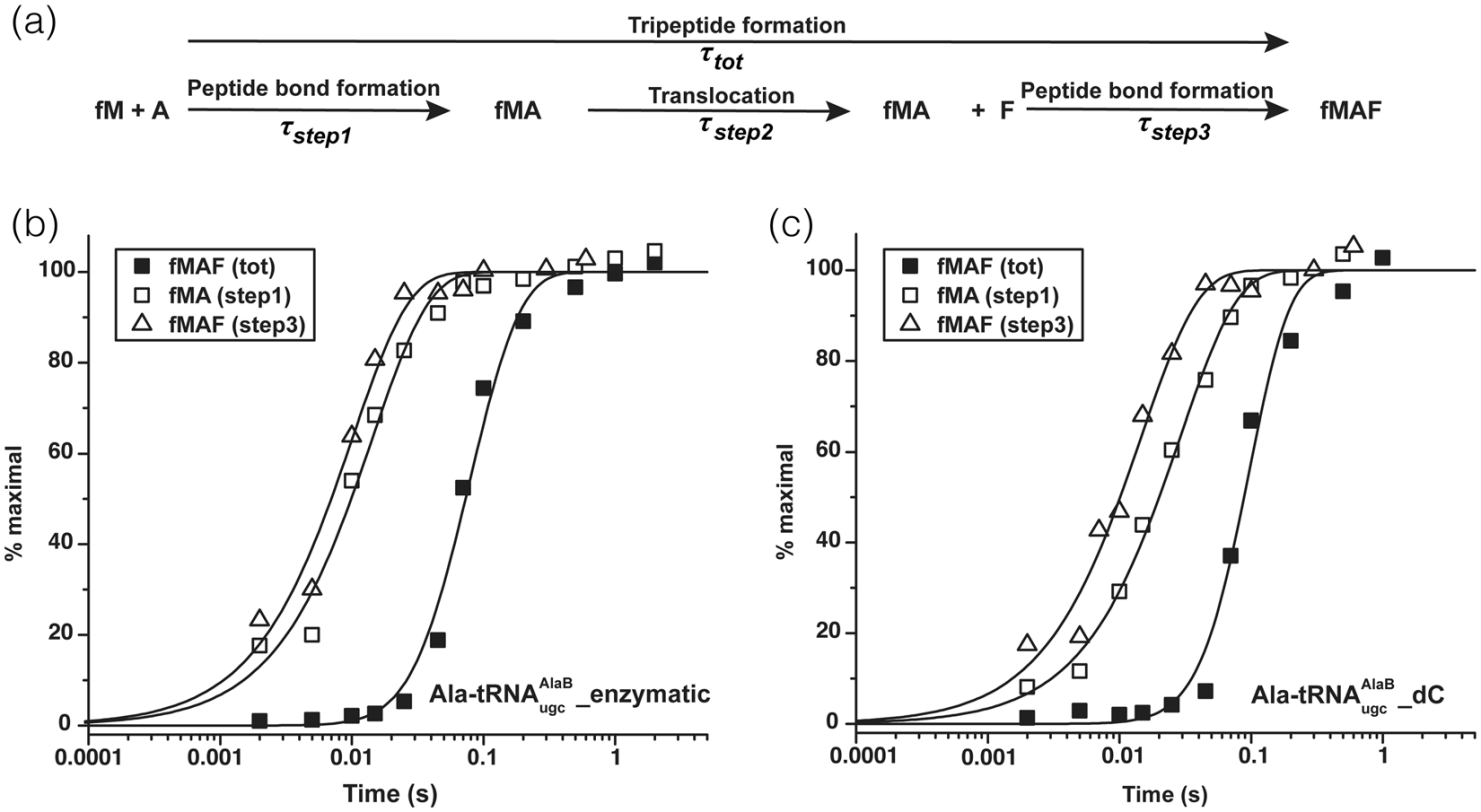
Assays for translocation of fMA-tRNA^Ala^ from ribosomal A- to P-site and for peptidyl donor activity. (**a**) Kinetic scheme of the assays. After determining *τ*_*tot*_, *τ*_*step1*_ and *τ*_*step3*_ independently in parallel, the mean time for translocation can be calculated as *τ*_*step2*_ = *τ*_*tot*_ − *τ*_*step1*_ − *τ*_*step3*_. Representative time courses for *τ*_*tot*_, *τ*_*step1*_ and *τ*_*step3*_ measurements with Ala-tRNA^AlaB^_ugc__enzymatic (**b**) and Ala-tRNA^AlaB^_ugc__dC (**c**) substrates are shown (see Table 2). Experiments were conducted at least in duplicates.

We next analyzed for effects on translocation of fMA-tRNA^Ala^ from the A to P site and the subsequent donor activity of the dipeptidyl-tRNA in peptidyl transfer. Experimentally, 3 reactions could be measured in parallel^26^ (Fig. 4a): (i) fMA formation from fMet-tRNA^fMet^ and Ala-tRNA^Ala^ (step1); (ii) fMAF tripeptide formation from fMet-tRNA^fMet^, Ala-tRNA^Ala^ and Phe-tRNA^Phe^ (tot); and (iii) fMAF tripeptide formation from translocated fMA-tRNA^Ala^ and Phe-tRNA^Phe^ (step3). The mean time for translocation from A site to P site could then be calculated as *τ*_*step2*_ = *τ*_*tot*_ − *τ*_*step1*_ − *τ*_*step3*_ (Fig. 4 and Table 2). The *τ*_*step1*_ for reaction with Ala-${{\rm{tRNA}}}_{{\rm{ugc}}}^{{\rm{AlaB}}}\_{\rm{dC}}$ (~29 msec) was larger than that when the tRNA was fully ribo-tRNA^Ala^ (~16 msec), as expected. Surprisingly, the *τ*_*step3*_ for the two cases had no significant difference (Table 2), indicating that the C75 2′H did not affect the peptidyl donor activity of the P site fMA-tRNA^Ala^ in the peptidyl transfer to Phe-tRNA^Phe^. Also unexpectedly, the calculated *τ*_*step2*_ for the two cases were similar, indicating that the penultimate dC did not affect translocation of fMA-tRNA^Ala^ from the ribosomal A site to the P site.

**Table 2:** Kinetic parameters for fMAF tripeptide formation (see Fig. 4).

AA-tRNA	*τ*_*tot*_ (msec)	*τ*_*step1*_ (msec)	*τ*_*step3*_ (msec)	*τ*_*step2*_ (msec)
Ala-${{\rm{tRNA}}}_{{\rm{ugc}}}^{{\rm{AlaB}}}\_{\rm{enzymatic}}$	90 ± 2	16 ± 1	13 ± 1	61 ± 2
Ala-${{\rm{tRNA}}}_{{\rm{ugc}}}^{{\rm{AlaB}}}\_{\rm{dC}}$	102 ± 8	29 ± 2	15 ± 2	58 ± 8

Mean values were calculated from at least two independent experimental results with their propagated standard deviations.

### The C75 2′H inhibits P- to E-site translocation

**Figure 5:**
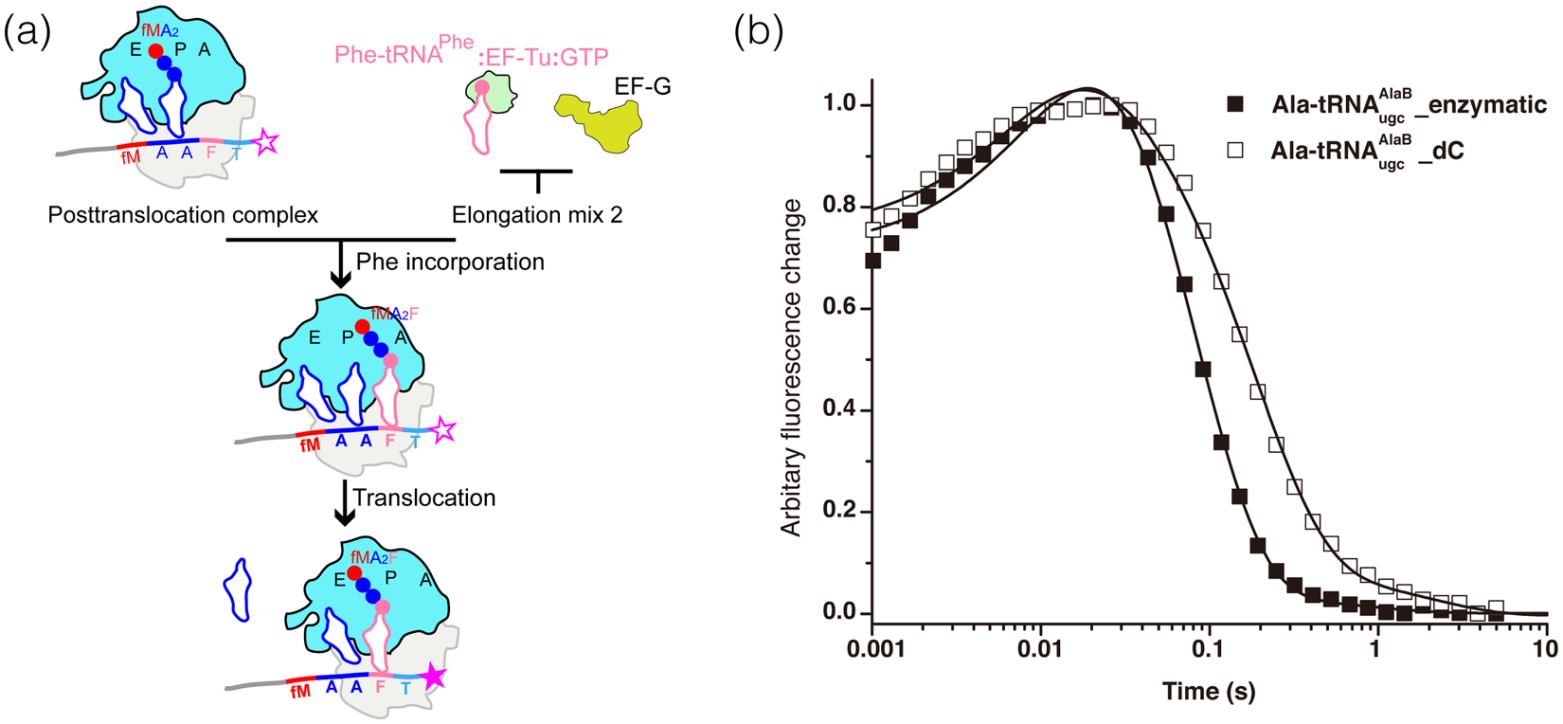
Assay of translocation of tRNA^Ala^ from ribosomal P- to E-site. (**a**) Schematic of stopped-flow experiment. Post-translocation complex programmed with 3′ pyrene-labeled mMAAFT and fMA_2_-tRNA^Ala^ in the P site was mixed with elongation mix 2 containing Phe-tRNA^Phe^ and EF-G on a stopped-flow apparatus. After the very fast fMA_2_F-tRNA^Phe^ formation, the tRNAs were translocated from A and P sites to P and E sites with concomitant moving of the 3′ end of the mRNA towards the ribosome, leading to quenching of the pyrene fluorophore fluorescence (shown as an open star turning to a filled star). (**b**) Comparison of the representative time courses of P- to E-site translocation of tRNA^Ala^ in the experiments done with Ala-tRNA^AlaB^_ugc__enzymatic (filled squares) and Ala-tRNA^AlaB^_ugc__dC (open squares). For better comparison, a digital filter was used to reduce the noise and the number of data points in the traces and time courses were normalized with the maximum value in each curve as 1 and the minimum value as 0. Solid lines represent the fitting of the traces to a double-exponential model. Experiments were conducted at least in duplicates (see text for standard deviations).

Finally we analyzed for effects on P- to E-site translocation of the P-site deacylated C75 2′H tRNA^Ala^. This step occurred 3 times during our incorporation of 5 consecutive Ala-tRNA^Ala^ substrates^11^. In order to isolate the effect of the C75 2′H on a single translocation step, we used a simpler translocation assay here: monitoring the mRNA movement with a 3′ pyrene-labeled mMA_2_FT mRNA^27, 28^. A post-translocation complex programmed with this fluorescent mRNA and fMA_2_-tRNA^Ala^ in the P site (and the same tRNA^Ala^ at the E site, if still bound) was rapidly mixed with EF-Tu:GTP:Phe-tRNA^Phe^ ternary complex together with EF-G:GTP on a stopped-flow apparatus (Fig. 5a). First, before translocation can occur, there is a very fast peptide bond formation. Then the deacylated P-site tRNA^Ala^ is translocated to the E site along with the mRNA movement. The monitored fluorescence signal is quenched with the 3′ pyrene label moving towards the ribosome (Fig. 5b)^27, 28^. The overall mean time for the fluorescence change is the sum of the mean times for the Phe-tRNA^Phe^ incorporation and EF-G-catalyzed mRNA movement. The overall mean time for Ala-${{\rm{tRNA}}}_{{\rm{ugc}}}^{{\rm{AlaB}}}\_{\rm{enzymatic}}$ was ~90 (91 ± 3) msec, compared with ~190 (189 ± 7) msec for Ala-${{\rm{tRNA}}}_{{\rm{ugc}}}^{{\rm{AlaB}}}\_{\rm{dC}}$. Assuming the incorporation mean time for Phe-tRNA^Phe^ was the same as measured in the step3 described above (~15 msec for both the cases, Table 2), translocating the tRNA carrying the C75 2′H from P site to E site would take ~175 msec. This is ~2-fold slower compared with the P- to E-site translocation of the fully ribo-tRNA (~75 msec)﻿ and also τ_*step2*_ values for rC and dC (﻿T﻿able 2)﻿. Again, this effect was not predicted from the crystal structures (Fig. 1e).

## Conclusion

By systematic kinetic analyses, we found that the C75 2′H in the A-site Ala-tRNA^Ala^_ugc_ impeded its peptidyl acceptor activity and the C75 2′H in the P-site deacylated tRNA^Ala^_ugc_ slowed translocation to the E site. This defines the mechanism by which this subtle atomic mutation in the tRNA^Ala^_ugc_ decreased the efficiencies of ribosomal polymerization. Delivery by EF-Tu was not significantly affected. Unexpectedly, at least according to our predictions from high-resolution co-crystal structures of full-length tRNA^fMet^ and tRNA^Phe^ analogs bound to the ribosome, the C75 2′H in tRNA^Ala^_ugc_ did not affect peptidyl donor activity or A- to P-site translocation. This suggests that, either the interpretations of the bona fide snap shots of the translation machinery cannot directly be applied to the systems when different tRNA substrates are used, or our kinetics approach can lead to higher resolution of the mechanisms of translation and thus can serve as a good complementary technology to the structural methods. Generalization of our conclusions will require follow up studies with other tRNAs. Nevertheless, our kinetics results define the first translational roles of a C75 2′OH in an *in vitro* tRNA transcript and expand our understanding of the importance of 2′OH groups in RNA function.

## Materials and Methods

### Materials

*N*-NVOC-Ala-pdCpA was prepared and characterized as before^29^. Synthetic 3′CA-truncated and full-length ${{\rm{tRNA}}}_{{\rm{ugc}}}^{{\rm{AlaB}}}$ were prepared as before^10^. After ligation of the *N*-NVOC-Ala-pdCpA to the 3′CA-truncated tRNA^AlaB^ transcript, *N*-NVOC-Ala-tRNA^AlaB^ was purified on a Q-column as described and the NVOC protecting group was removed by photolysis^30^. Synthetic mRNA, mMAF, was prepared from *in vitro* transcription with DNA template made from synthetic oligos^31^ and the sequence was 5′ gggaauucgggcccuuguuaacaauuaaggagguauauc**auggcauuu**uaauugcagaaaaaaaaaaaaa 3′ with the coding region in bold (the processivity of ribosomal polymerization using mRNA transcribed from synthetic oligos was indistinguishable from that using mRNA from cloned plasmid based on kinetics with mMAAAAA^11^). The over-expressed *E. coli* tRNA^fMet^ and tRNA^Phe^ were prepared respectively as described^32, 33^. All other reagents were prepared as described^34^.

### Kinetics of dipeptide synthesis at different ternary complex concentrations

To compare the A site acceptor reactivity of Ala-${{\rm{tRNA}}}_{{\rm{ugc}}}^{{\rm{AlaB}}}\_{\rm{dC}}$ with that of Ala-${{\rm{tRNA}}}_{{\rm{ugc}}}^{{\rm{AlaB}}}\_{\rm{enzymatic}}$, fMet-Ala (fMA) dipeptide formation mean times at different ternary complex concentrations were measured for both substrates. The *in vitro* translation reactions were at 37 °C in pH 7.5 polymix buffer supplemented with 1 mM ATP, 1 mM GTP, 10 mM PEP, 1 μg/mL pyruvate kinase and 0.1 μg/mL myokinase for energy regeneration^34^. Two mixtures were prepared on ice in the polymix buffer. A ribosomal mix contained MRE600 *E. coli* 70 S ribosomes (1 μM), IF1 (1.5 μM), IF2 (0.5 μM), IF3 (1.5 μM), [^3^H]fMet-tRNA^fMet^ (2 μM), and mMAF (4 μM). For the case of Ala-${{\rm{tRNA}}}_{{\rm{ugc}}}^{{\rm{AlaB}}}\_{\rm{dC}}$, a ternary complex mix contained 30 μM EF-Tu, 3 μM EF-Ts and different concentrations (2, 4, 8 and 12 μM) of Ala-${{\rm{tRNA}}}_{{\rm{ugc}}}^{{\rm{AlaB}}}\_{\rm{dC}}$. For the case of Ala-${{\rm{tRNA}}}_{{\rm{ugc}}}^{{\rm{AlaB}}}\_{\rm{enzymatic}}$, different concentrations (2, 4, 8 and 12 μM) of the full-length transcript ${{\rm{tRNA}}}_{{\rm{ugc}}}^{{\rm{AlaB}}}$, 0.4 mM alanine and 0.4 unit/μL AlaRS were added to the ternary complex mix instead of the AA-tRNA. The mixtures were preincubation at 37 °C for 15 min. Then equal volumes of the ribosomal mix and the ternary complex mix were rapidly mixed in a temperature-controlled quench-flow apparatus (RQF-3; KinTeck Corp.). The reaction was quenched with final 17% formic acid after different reaction times. Samples were centrifuged at 20,000 × g for 15 min at 4 °C and the supernatant was discarded. To the pellets, 120 μL 0.5 M KOH was added and samples were incubated at 37 °C for 10 min to deacylate the tRNAs. Then formic acid was added to 17% to precipitate the deacylated tRNAs. After another 10 min incubation on ice, samples were centrifuged at 20,000 × g for 15 min at 4 °C. Supernatant containing [^3^H] peptides and the unreacted [^3^H]fMet from each time point was analyzed by C18 reverse phase HPLC coupled with a β-RAM model 3 radioactivity detector (IN/US Systems). The separation of fMet and fMA dipeptide was done by elution with 0% methanol/99.9% H_2_O/0.1% trifluoroacetic acid for 20 min at 1 mL/min followed by another 40 min at 0.45 mL/min. After quantifying the [^3^H] fMA fraction from the total [^3^H] signal at each time point, the data were analyzed by the nonlinear regression program Origin 7.5 with a 2-step kinetic model^31^ and dipeptide formation reaction mean times, *τ*_*dip*_, at each ternary complex concentration were obtained. The inverses of the mean times were then plotted against ternary complex concentrations and fitted to the hyperbolic Michaelis-Menten equation to deduce the maximal overall rate constant for dipeptide formation, *k*_*cat*_, as well as *K*_*M*_ values^34^.

### Simultaneous measurement of GTP hydrolysis and dipeptide synthesis

To simultaneously measure the mean times for GTP hydrolysis on EF-Tu (*τ*_*GTP*_) and dipeptide formation (*τ*_*dip*_), a ribosomal mix was prepared similarly to that described above, except that the 70S ribosome was added to 2 μM and [^3^H]fMet-tRNA^fMet^ to 2.5 μM. In the ternary complex mix, unlabeled GTP was omitted and ATP was added to 2 mM. The ternary complex mix contained 1 μM [^3^H]GTP, 1 μM EF-Tu and 1.2 μM Ala-${{\rm{tRNA}}}_{{\rm{ugc}}}^{{\rm{AlaB}}}\_{\rm{dC}}$ or 0.4 mM alanine, 0.4 unit/μL AlaRS and 1.2 μM full-length transcript Ala-${{\rm{tRNA}}}_{{\rm{ugc}}}^{{\rm{AlaB}}}\_{\rm{enzymatic}}$. Sample treatment and data analysis were done in the same way as described^31^. The overall mean time, *τ*_*acc,pep*_, for all the steps that lead to peptide bond formation after GTP hydrolysis on EF-Tu was obtained by subtracting the mean time for GTP hydrolysis on EF-Tu from the mean time for dipeptide formation.

### A- to P-site translocation assay

To measure the rate of the translocation step between the formation of fMA dipeptide and the formation of fMet-Ala-Phe (fMAF) tripeptide, two reactions were done in parallel^27^. The first reaction was fMAF tripeptide synthesis starting from 70S initiation complex such that the mean times for fMA dipeptide formation (*τ*_*step1*_) and fMAF tripeptide formation (*τ*_*tot*_) could be deduced. In this reaction, the ribosomal mix contained 0.6 μM 70S ribosome, 0.9 μM IF1, 0.3 μM IF2, 0.9 μM IF3, 1 μM mMAF and 0.5 μM [^3^H]fMet-tRNA^fMet^. The ternary complex mix contained 20 μM EF-Tu, 2 μM EF-Ts, 22 μM EF-G, 0.4 mM phenylalanine, 0.4 unit/μL PheRS, 8 μM tRNA^Phe^ and 6 μM Ala-${{\rm{tRNA}}}_{{\rm{ugc}}}^{{\rm{AlaB}}}\_{\rm{dC}}$ or 0.4 mM alanine, 0.4 unit/μL AlaRS and 6 μM full-length transcript ${{\rm{tRNA}}}_{{\rm{ugc}}}^{{\rm{AlaB}}}$. The reaction and sample treatment were performed in the same way as described above for the dipeptide synthesis experiment. In the second reaction, fMAF tripeptide was synthesized from the posttranslocation complex that had the fMA-tRNA in the P site, such that the second peptide bond formation mean time, *τ*_*step3*_, could be obtained. In this reaction, three mixtures were prepared. The ribosomal mix had doubled concentrations of the components as in the ribosomal mix in the first reaction. An elongation mix, **E1**, contained 8 μM EF-Tu, 1 μM EF-Ts, 4 μM EF-G and 12 μM Ala-${{\rm{tRNA}}}_{{\rm{ugc}}}^{{\rm{AlaB}}}\_{\rm{dC}}$ or 0.4 mM alanine, 0.4 unit/μL AlaRS and 12 μM full-length transcript ${{\rm{tRNA}}}_{{\rm{ugc}}}^{{\rm{AlaB}}}$. The third mix, **E2**, contained 16 μM EF-Tu, 1.5 μM EF-Ts, 20 μM EF-G, 0.4 mM phenylalanine, 0.4 unit/μL PheRS and 8 μM tRNA^Phe^. After preincubation for 15 min at 37 °C for the three mixtures, equal volumes of the ribosomal mix and **E1** mix were rapidly mixed in the reaction tube for 5 sec before incubation on ice for 10 min. The resulting mixture and **E2** mix were applied to the quench-flow apparatus for kinetic experiments. The reaction and sample treatment were performed in the same way as described above. Dipeptide, tripeptide and unreacted fMet was separated on C18 reverse phase HPLC by eluting with 0% methanol/99.9% H_2_O/0.1% trifluoroacetic acid for 20 min at 1 mL/min and another 10 min at 0.45 mL/min, followed by 30 min elution with 58% methanol/42% H_2_O/0.1% trifluoroacetic acid at 0.45 mL/min. Data analysis was done as described^26^ and the complete translocation time, *τ*_*step2*_, was calculated as *τ*_*step2*_ = *τ*_*tot*_ − *τ*_*step1*_ − *τ*_*step3*_.

### P- to E-site translocation rate measurement

The 3′ pyrene-labeled mMA_2_FT mRNA was chemically synthesized by IBA GmbH (Göttingen), kindly provided M. Holm and S. Sanyal, and had the sequence 5′ uaacaauaaggagguauuaa**auggcagcauuuacg** 3′. Three mixtures were prepared for this measurement similar to the three mixtures in the A- to P- site translocation assay. A ribosomal mix contained 0.8 μM 70S ribosome, 1.2 μM IF1, 0.4 μM IF2, 1.2 μM IF3, 1 μM [^3^H]fMet-tRNA^fMet^ and 1.5 μM pyrene-labeled mRNA. An elongation mix, **E1**, contained 2 μM EF-Tu, 0.25 μM EF-Ts, 4 μM EF-G and 2 μM Ala-${{\rm{tRNA}}}_{{\rm{ugc}}}^{{\rm{AlaB}}}\_{\rm{dC}}$ or 0.2 mM alanine, 0.2 unit/μL AlaRS and 2 μM full-length transcript ${{\rm{tRNA}}}_{{\rm{ugc}}}^{{\rm{AlaB}}}$. The third mix, **E2**, contained 10 μM EF-Tu, 0.5 μM EF-Ts, 38 μM EF-G, 0.4 mM phenylalanine, 0.4 unit/μL PheRS and 6 μM tRNA^Phe^. After preincubation of the three mixtures for 15 min at 37 °C, equal volumes of the ribosomal mix and **E1** mix were rapidly mixed in the reaction tube for 10 sec before incubation on ice for 10 min. The resulting mixture and **E2** mix were loaded onto a stopped flow instrument (Applied Photophysics SX20). Pyrene fluorescence was excited at 343 nm and the change in fluorescence after mixing was recorded using a 360 nm long pass filter (Newport 10CGA-360). Data analysis was done as described^27^.

### Data availability

All data generated or analyzed during this study are included in this published article (and its Supplementary Information files).

## Electronic supplementary material

Supplementary Information
