# Supplementary material for: Translational roles of the C75 2′OH in an in vitro tRNA transcript at the ribosomal A, P and E sites
Source: Sci Rep. 2017 Jul 27;7:6709. doi: 10.1038/s41598-017-06991-6 (PMC5532260; doi:10.1038/s41598-017-06991-6)
Supplement: Supplementary file 1 — Supplementary Information [file 41598_2017_6991_MOESM1_ESM.pdf]

Supplementary Information

Translational roles of the C75 2'OH in an in vitro tRNA transcript at the ribosomal A, P and E sites

Jinfan Wang and Anthony C. Forster*

Department of Cell and Molecular Biology, Uppsala University, Husargatan 3, Box 596, Uppsala 75124, Sweden

*Corresponding Author
A.C. Forster, tel: +46-18-4714618, e-mail: a.forster@icm.uu.se

Contents:

Supplementary Table 1

Supplementary Figures 1 and 2

Supplementary Table 1.
Kinetic values for dipeptide synthesis from fMet-tRNA^{fMet} and Ala-tRNAs at different ternary complex concentrations*

**Ala-tRNA^{Ala}**	**[Ternary complex] (μM)**	**k_{dip} (s^{-1})**
Ala-tRNA$^{AlaB}_{ugc}$_enzymatic	1	31.6 ± 3.8
	2	56.1 ± 5.0
	4	82.5 ± 9.6
	6	93.1 ± 11.4
Ala-tRNA$^{AlaB}_{ugc}$_dC	1	14.3 ± 0.8
	2	19.8 ± 1.0
	4	32.3 ± 1.5
	6	37.0 ± 1.8

* Mean values were calculated from at least two independent experimental results with their propagated standard deviations.

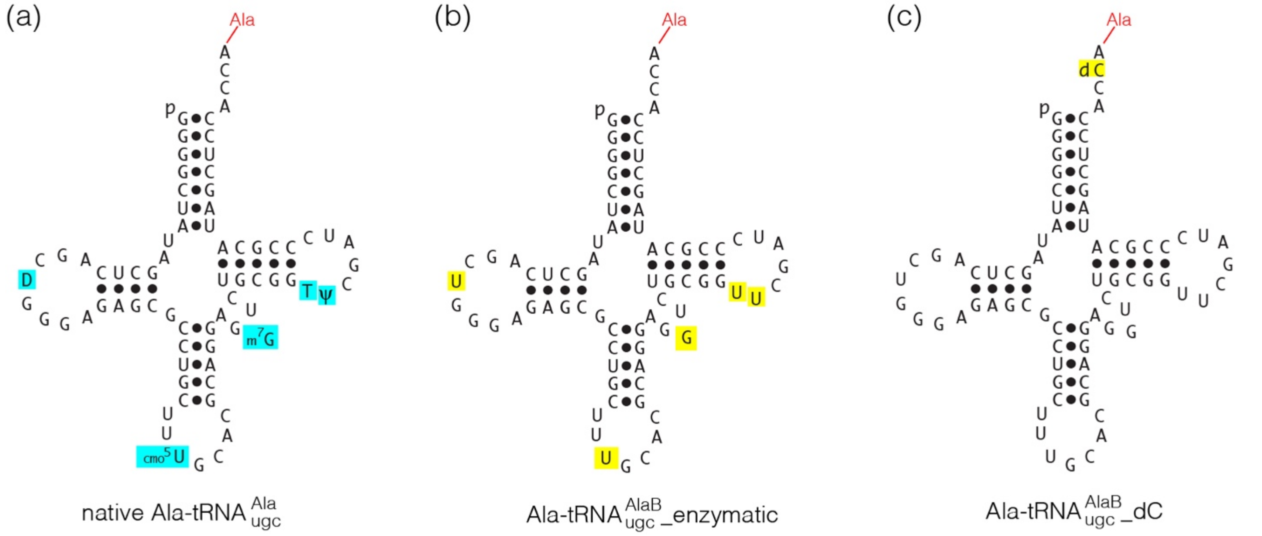

Supplementary Figure 1. Secondary structures of the Ala-tRNAs. (a) Native Ala-tRNA^{Ala} is shown with post-transcriptional modifications highlighted with a cyan background and the AA in red. (b) Ala-tRNA$^{AlaB}_{ugc}$_enzymatic was prepared by charging *in vitro*-synthesized, unmodified, tRNA^{Ala} transcript with Ala by AlaRS. The changes from the native sequence are highlighted with yellow. (c) Ala-tRNA$^{AlaB}_{ugc}$_dC was prepared by ligating *N*-NVOC-Ala-pdCpA to *in vitro*-synthesized, 3'-CA-truncated, tRNA^{Ala} transcript using T4 RNA ligase followed by photolytic removal of the NVOC amino protecting group. The difference between Ala-tRNA$^{AlaB}_{ugc}$_dC and Ala-tRNA$^{AlaB}_{ugc}$_enzymatic is the dC75 highlighted with yellow.

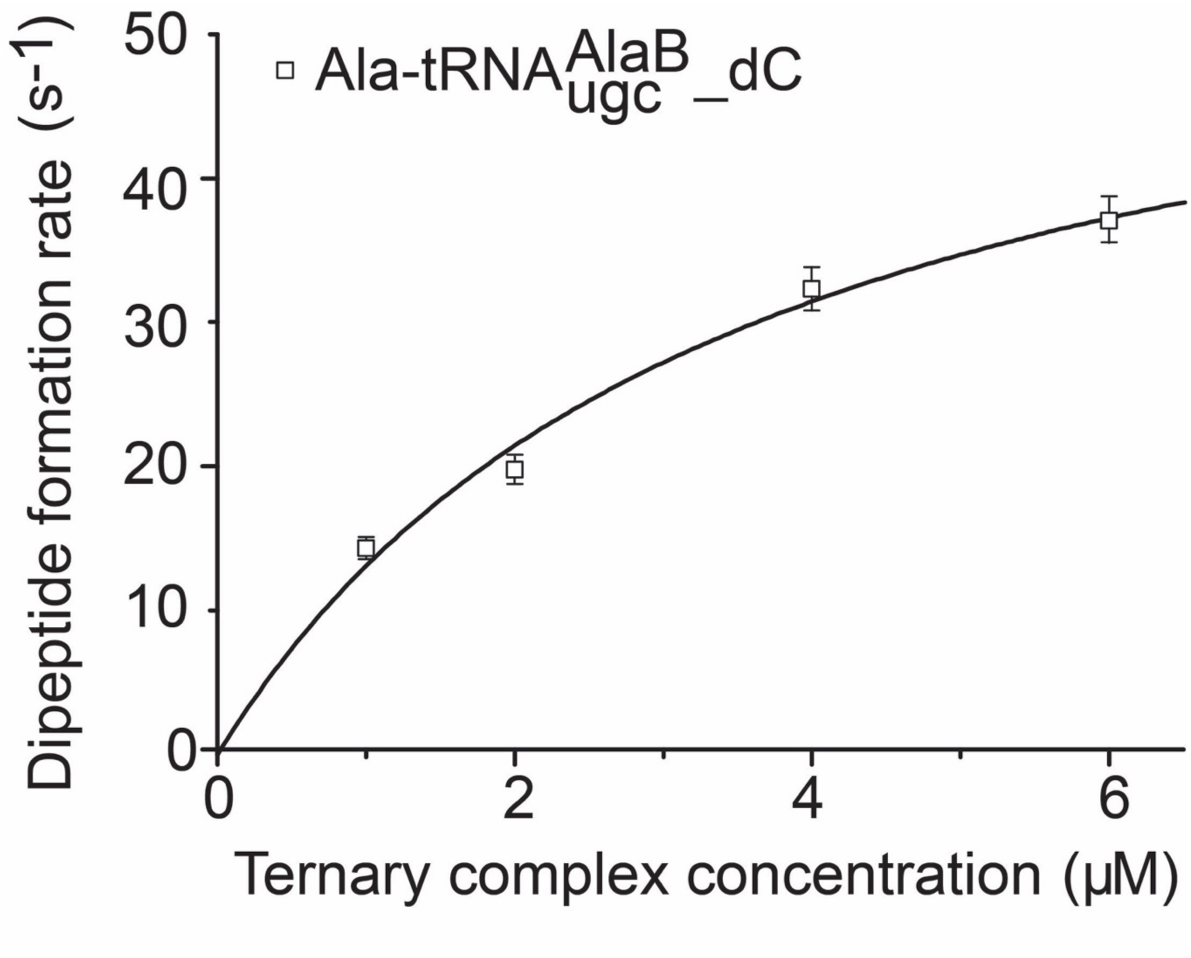

Supplementary Figure 2. The rates of dipeptide formation from fMet-tRNA^{fMet} and Ala-tRNA$^{AlaB}_{ugc}$_dC versus ternary complex concentrations (same as in Figure 3a) is replotted here for a better visualization of the error bars.
